# Visual Input Is the Main Trigger and Parametric Determinant for Catch-Up Saccades During Video Head Impulse Test in Bilateral Vestibular Loss

**DOI:** 10.3389/fneur.2018.01138

**Published:** 2019-01-04

**Authors:** Christian Van Nechel, Alionka Bostan, Ulla Duquesne, Charlotte Hautefort, Michel Toupet

**Affiliations:** ^1^Unité Troubles de l'Equilibre et Vertiges, Centre Hospitalier Universitaire Brugmann, Brussels, Belgium; ^2^Unité de Neuro-Ophtalmologie, Hôpital Erasme, Brussels, Belgium; ^3^Institut de Recherche Oto-Neurologique (IRON), Paris, France; ^4^Clinique des Vertiges, Brussels, Belgium; ^5^Service ORL, APHP CHU Lariboisière, Paris, France; ^6^Centre d'Explorations Fonctionnelles Otoneurologiques, Paris, France

**Keywords:** vestibulo-ocular reflex, catch-up saccades, bilateral vestibulopathy, video head impulse test, covert saccade, overt saccade, dynamic vision

## Abstract

Patients with vestibular deficit use slow eye movements or catch-up saccades (CUS) to compensate for impaired vestibulo-ocular reflex (VOR). The purpose of CUS is to bring the eyes back to the visual target. Covert CUS occur during high-velocity head rotation and overt CUS are generated after head rotation has stopped. Dynamic visual acuity is improved with an increased rate and gain of CUS. Nevertheless, the trigger and the parametric determinants of CUS are still under debate. To clarify the underlying mechanism, especially the visual contribution, we analyzed the number, amplitude and latencies of the CUS in relation with the extent of VOR deficiency. The head and eye movements were recorded in 17 patients with bilateral vestibular loss (BVL) and in 33 subjects with normal VOR gain using the Video Head Impulse Test (vHIT) in two conditions: with visible target and in darkness with an imaginary target. Our study shows that in darkness without visible target the number of CUS is significantly reduced and the relationship between the amplitude of CUS and gaze position error is lost. Results showed that there is a correlation between the number of CUS and the drop in VOR gain. CUS occurring during the head movement and when the head remained still were not always sufficiently accurate. Up to four consecutive CUS could be required to bring eyes back to the visible target. A positive correlation was found between the amplitude of overt saccades with visible target and the gaze position error, namely the remaining eye movement to reach the target. These results suggest that the visual inputs are the main trigger and parametric determinant of the CUS or at least the presence of a visual target is necessary in most cases for a CUS to occur.

## Introduction

One of the main mechanisms used by humans to keep a visual target on the fovea during head movements is the vestibulo-ocular reflex (VOR). The VOR moves the eyes in the direction opposite to head movement with a ratio between eye velocity and head velocity close to −1. In bilateral loss of vestibular function, the patient is unable to maintain the gaze on target during fast head movements and may experience oscillopsia, when he gets the illusion of unstable objects in the visual field. The eyes are initially carried away with the head movement, then one or several corrective saccades occur, bringing the image back on the fovea. Such saccades act as a compensatory, refixation mechanism, they are regarded as catch-up saccades (CUS). CUS have also been described during and after transient high velocity head rotations in patients with unilateral vestibular loss (1).

Two types of CUS have been described. Covert CUS occur early, while the head is still moving, most likely imperceptible by the examiner; overt CUS occur once the head impulse has stopped, visible by the observer (2). The simple bedside head impulse test allows the detection of overt CUS only (3). With the help of the search-coil recording and video head impulse test (vHIT) both types of CUS can be detected and analyzed (4).

In most cases of unilateral or bilateral VOR deficit, both types of CUS are found. Some patients present only one type (5) or even none if they blink, have a relative high VOR gain or move the head too slowly (6). CUS may also occur in subjects with normal VOR gain and their frequency increases with age (7).

There is a great disparity in the literature about the latencies of these CUS, from about 70 ms (5, 6) to 150 ms (5, 8). The trigger and parametric determinants of the CUS are still under debate. Conceptually, the relationship between the amplitude of CUS and the gaze position error (GPE) (6) could be determined by several factors, such as the residual or contralateral vestibular function, visual input or combined input from both oculomotor and cervical proprioception. The relatively short latency led some authors to suggest that an accurate CUS cannot be attributed to vision and is driven by vestibular input in unilateral vestibular deficit (6, 9). After bilateral neurectomy, the disappearance of CUS when the target is switched-off 1 s before the head impulse led other authors to promote a crucial role of the visual input for the accuracy of CUS (10). Nevertheless, Lehnen et al. (10) found similar CUS latencies in one patient with residual vestibular function compared to patients with complete bilateral vestibular loss (BVL), suggesting that residual vestibular function does not modify the triggering delay of CUS in the light. But, this patient performed efficient CUS with similar latencies in darkness and in light, suggesting that residual vestibular function provides a major contribution in the generation of the CUS in darkness.

In our practice, we observed that CUS are less accurate in bilateral than unilateral vestibular loss and some patients showed more than one CUS after the end of the head movement. This suggested that in BVL, the first overt saccades are not always accurate enough to bring the eyes back on the visual target. As shown by Weber, the amplitude of subsequent saccades becomes smaller (11). Even if CUS by themselves could not improve vision during the head movement, their occurrence is correlated with an improvement of the dynamic visual acuity (8). The preservation of the static visual acuity during head movement requires a stable image (retinal slip < 4°/s) for more than 50 ms (12). The visual acuity declines progressively from the fovea out to the periphery of the retina. Early CUS bring the target image closer to the fovea. In doing so, they reduce the blurred vision and diminish the time needed to reacquire the target on the fovea (13) at the end of the head thrust. However, they cannot prevent the retinal slip which degrades the vision during a high velocity head movement. Ramaioli et al. (14) showed that the occurrence of early CUS may improve dynamic visual acuity, but the visual stimulus remained displayed when the head velocity decreased under 80°/s, allowing the eye smooth pursuit to suppress the residual retinal slip.

Head movements only rehabilitation technique has been suggested to improve dynamic vision for BVL by an increase in head impulse gain and/or an increase in compensatory saccade amplitude (15). This heterogeneity requires further insights into the mechanism triggering CUS to identify interventions promoting their occurrence for the rehabilitation of patients with BVL.

The aim of this study was to identify which factors determine the parameters of these CUS in patients with complete or partial bilateral vestibular deficit. Therefore, vHIT was performed in subjects with either BVL or normal VOR gain in standard conditions (visible target in lighted room) and in total darkness with an imaginary target in order to evaluate the influence of visual suppression on VOR gain and associated CUS.

## Materials and Methods

### Participants

The study included a first group of 17 patients with BVL. BVL was mostly idiopathic in 14, caused by gentamycin toxicity in one, bi-lateralization of Menière's disease in one, and acute unilateral peripheral vestibular loss followed later by another attack on the other side in one patient.

These patients were aged between 29 and 80 years (mean 62 ± 12.9 years). The BVL was assessed based on a sum under 20°/s for the maximum slow phase velocities of the nystagmus induced by the caloric tests (30 s irrigation of 150–200 cm^3^ at 30°C and 44°C) (16), and non-identifiable responses to rotatory chair test. The inclusion criteria are in accordance with the diagnosis criteria consensus of the Barany Society (17). All of them were diagnosed several years before testing (8 years on average) and were in an intensive vestibular rehabilitation program, including gaze stabilization exercises.

The second group included 35 patients who presented with vertigo or dizziness and showed normal horizontal VOR gain (>0.8) at the vHIT. These patients were aged between 17 and 92 years (mean 50 ± 14.7 years). The diagnosis was vestibular migraine (15 patients), persistent perceptual postural dizziness (6 patients), benign paroxysmal positional vertigo (5 patients), space and motion discomfort (3 patients), motion sickness (2 patients), cervical canal stenosis (1 patient), polyneuropathy (1 patient), lacunar syndrome (1 patient) and vitreous floaters (1 patient).

All the patients gave written informed consent. The study was conducted according to the Helsinki declaration. The experimental protocol was approved by the local Ethics Committee (CCPPRB Paris).

### Data Collection

Head impulses were recorded with the ICS Impulse ver. 4.0^®^ vHIT (Otometrics A/S, Taastrup, Denmark). Calibration instructions were given for each patient before the test. During calibration, the subject kept the head still while switching the gaze between two laser dots on each side of a target through a small angle about 10°, to ensure the overlapping of head and eye movements. Horizontal head impulses to each side were manually delivered with unpredictable timing and direction by the physician, standing behind the subject. At least eight accepted head impulses, with an amplitude about 10°, head velocity about 200°/s and acceleration about 2000°/s2 were collected for each horizontal canal in each session.

For the first session, patients were instructed to fixate a red dot on a wall about 140 cm away from their sitting position in light (light-test).

In order to address the visual contribution to the VOR, at the end of the first session, similar head impulses were applied with the patient in total darkness, asking them to fixate an imaginary target that would be in the same position on the wall as during the test in light conditions (darkness-test). This was done because preliminary tests performed in dark conditions without any instruction for the patient gave invalid results owing to erratic eye movements. Total darkness was achieved using a vision-denied solution cup for the recorded right eye and an opaque patch for the left eye that were applied on Otometrics goggles in a completely darkened testing room. The vision-denied cup, which allows infrared light to pass while blocking light in the visible spectrum was provided by Otometrics.

### Data Analysis

**Figure 1:**
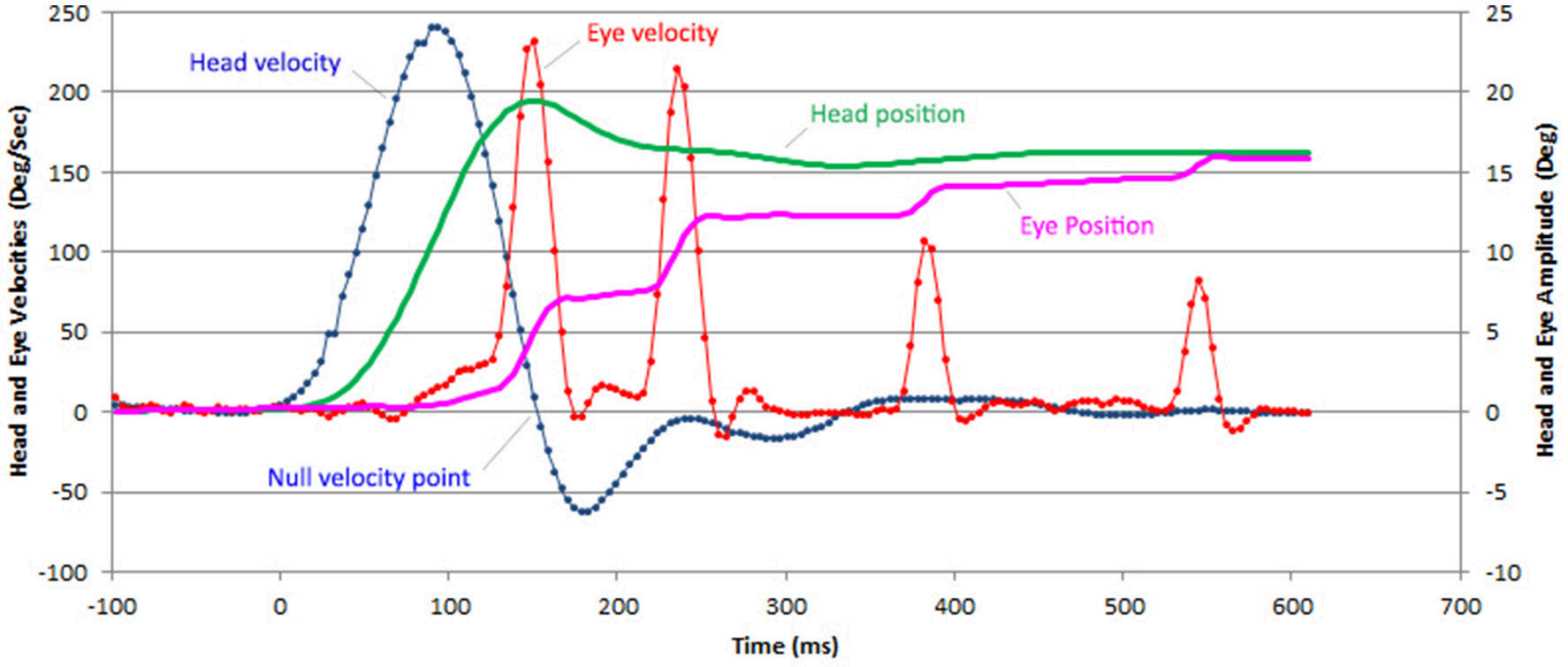
Example of a horizontal vHIT recording with 4 CUS in a patient with BVL. The head (green line) and eye (purple line) position are computed from the head (blue) and eye (red line) velocity data provided by the recording device (ICS Impulse Otometrics). Only saccades that bring the eyes closer to the target are considered catch-up saccades. The onset of all latencies was defined when head velocity reached 5°/s.

The gain values of the left and right horizontal VOR were used from the Otometrics ICS Impulse ver. 4.0^®^ software. Raw data from Otometrics software were exported and further analyzed through algorithms implemented in Microsoft Excel software. These algorithms define the head and eye velocities and positions over time as well as the latency, velocity and amplitude of CUS. This allowed us to determine the contribution of each CUS to attain the eye position to target position (Figure 1). Only CUS that brought the eye toward the target position were analyzed, with a maximum of four saccades in a limited acquisition time interval of 800 ms. Saccades were identified by their peak velocity. The onset of the first saccade was identified manually on the velocity trace or on the cumulative amplitude curve. As shown in Figure 1, in case of low VOR gain, this onset is most often easy to identify. The eye end position of each catch up saccade is settled 20 ms after its peak velocity. The saccade amplitude is the difference between this eye end position and the eye end position of the previous saccade. A preliminary manual analysis has shown that the eye position 20 ms after the peak velocity provides a reliable value to determine the saccade amplitude. The relative amplitude of CUS was defined as the ratio between the amplitude of the CUS and the head rotation amplitude at the end of the CUS. Relative gaze position error was defined at the end of each CUS as the ratio between the cumulative amplitudes of eye movement to the final amplitude of the head movement. We defined the latency onset as the instant when head velocity was >5°/s. We measured the maximum velocity latency for all CUS (*n* = 628) from the beginning of the head movement for the first CUS and from the latency of the previous CUS for the following CUS. Statistical analysis of the data was done using Dell Inc Statistica 13 and Microsoft Excel 1807 software. Student test was used to compare horizontal VOR gains in light vs. darkness. The maximum velocity latency distribution in light and darkness were analyzed by the Shapiro-Wilk test. The comparison of the CUS latency in light vs. darkness was performed using a Mann-Whitney test. The relation between the number of CUS and the VOR gain were evaluated with ANOVA test. The number of CUS in light and darkness were compared with a Chi-squared test.

## Results

### BVL Group

**Figure 2:**
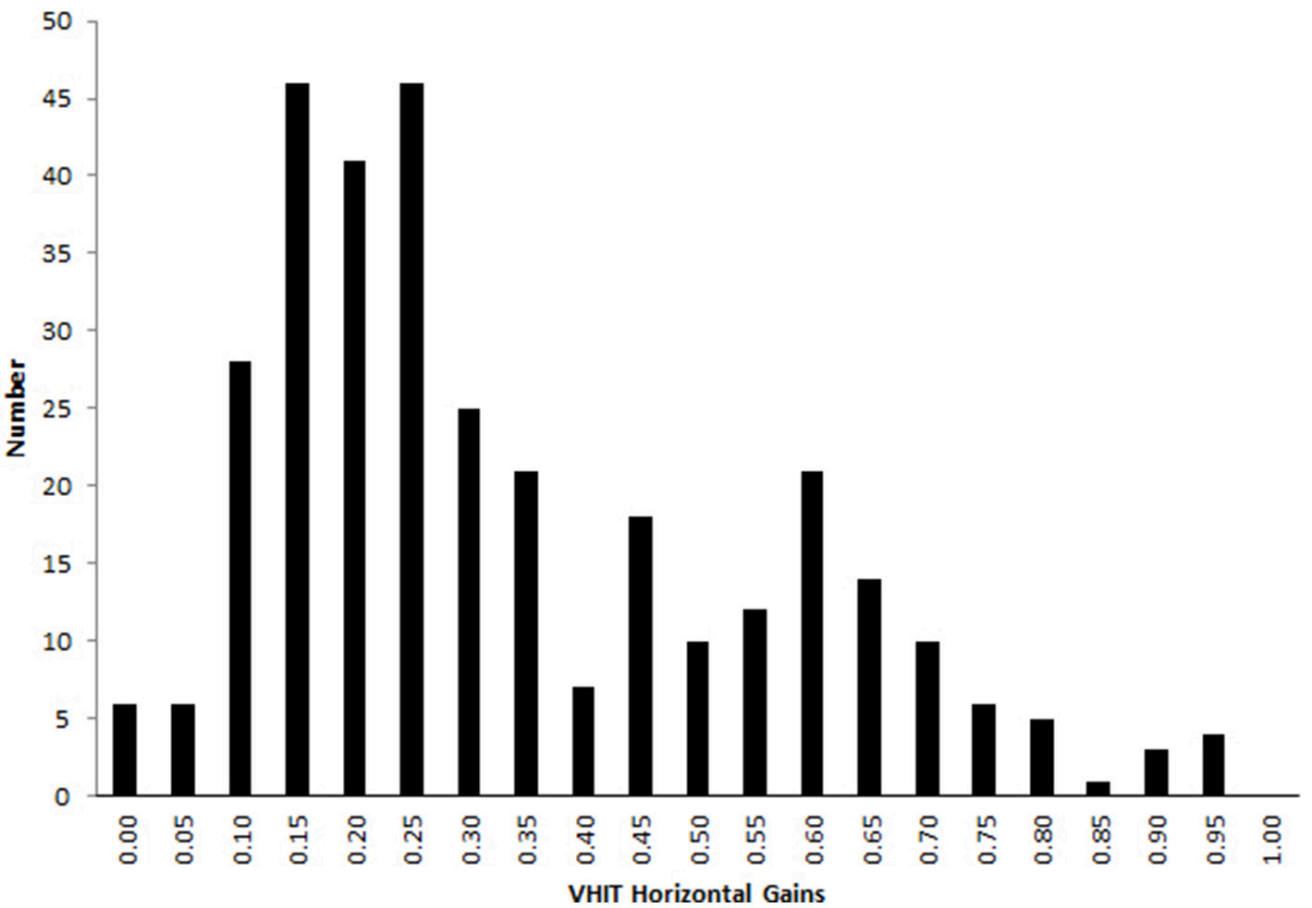
Horizontal VOR gain during vHIT with visible target in the bilateral vestibular deficient patients group. A proportion of 23 % (76 out of 330) head impulses showed a VOR gain over 0.5 and 4% (13 out of 330) over 0.8.

A total of 329 head impulses were delivered with light target and 319 with imaginary target. For light-test, average vHIT gains of right and left horizontal VOR were 0.32 ± 0.18 (range 0.08–0.79) (Table 1). Six out of 17 patients (80 light-test recordings) showed records with a VOR gain over 0.5 (24%) (Figure 2). For darkness-test, the horizontal VOR gains were significantly reduced: 0.27 ± 0.16 (range 0−0.9) (Student test for paired values, *p* = 0.003).

**Table 1:** The vHIT gains mean (range) collected with visible target and in darkness in the BVL group of 17 patients.

**Patient**	**vHIT gAINS**			
	**Visible target**		**Darkness**	
	**Left**	**Right**	**Left**	**Right**
1	0.59 (0.55–0.64)	0.39 (0.21–0.64)	0.59 (0.32–0.68)	0.31 (0.11–0.56)
2	0.24 (0.12–0.41)	0.27 (0.19–0.31)	0.35 (0.07–0.46)	0.22 (0.12–0.35)
3	0.49 (0.42–0.72)	0.69 (0.57–0.73)	0.43 (0.24–0.73)	0.58 (0.4–0.83)
4	0.58 (0.16–0.67)	0.49 (0.11–0.61)	0.61 (0.06–0.9)	0.44 (0–0.54)
5	0.16 (0.07–0.34)	0.12 (0–0.26)	0.18 (0–0.41)	0.14 (0–0.25)
6	0.30 (0.19–0.69)	0.17 (0.11–0.27)	0.22 (0–0.61)	0.27 (0.06–0.56)
7	0.58 (0.06–0.78)	0.30 (0.15–0.45)	0.28 (0.03–0.6)	0.32 (0.23–0.44)
8	0.08 (0–0.2)	0.17 (0.1–0.28)	0.07 (0–0.26)	0.10 (0–0.2)
9	0.16 (0.03–0.39)	0.22 (0.15–0.27)	0.11 (0–0.43)	0.16 (0–0.3)
10	0.40 (0.15–0.45)	0.35 (0–0.76)	0.34 (0.13–0.5)	0.27 (0.14–0.36)
11	0.17 (0.1–0.34)	0.25 (0.01–0.38)	0.17 (0.15–0.19)	0.24 (0.13–0.28)
12	0.16 (0.05–0.57)	0.19 (0–0.63)	0.29 (0–0.56)	0.17 (0–0.6)
13	0.52 (0.22–0.69)	0.50 (0.23–0.62)	0.51 (0.2–0.61)	0.48 (0.21–0.61)
14	0.28 (0.19–0.48)	0.30 (0.19–0.91)	0.23 (0.12–0.73)	0.14 (0–0.31)
15	0.32 (0.08–0.5)	0.79 (0.09–0.94)	0.28 (0.14–0.58)	−0.12 (0–0.63)
16	0.11 (0.09–0.16)	0.14 (0.1–0.24)	0.35 (0.08–0.69)	0.33 (0.12–0.55)
17	0.14 (0.09–0.18)	0.22 (0.17–0.27)	0.09 (0–0.14)	0.20 (0.09–0.38)

**Figure 3:**
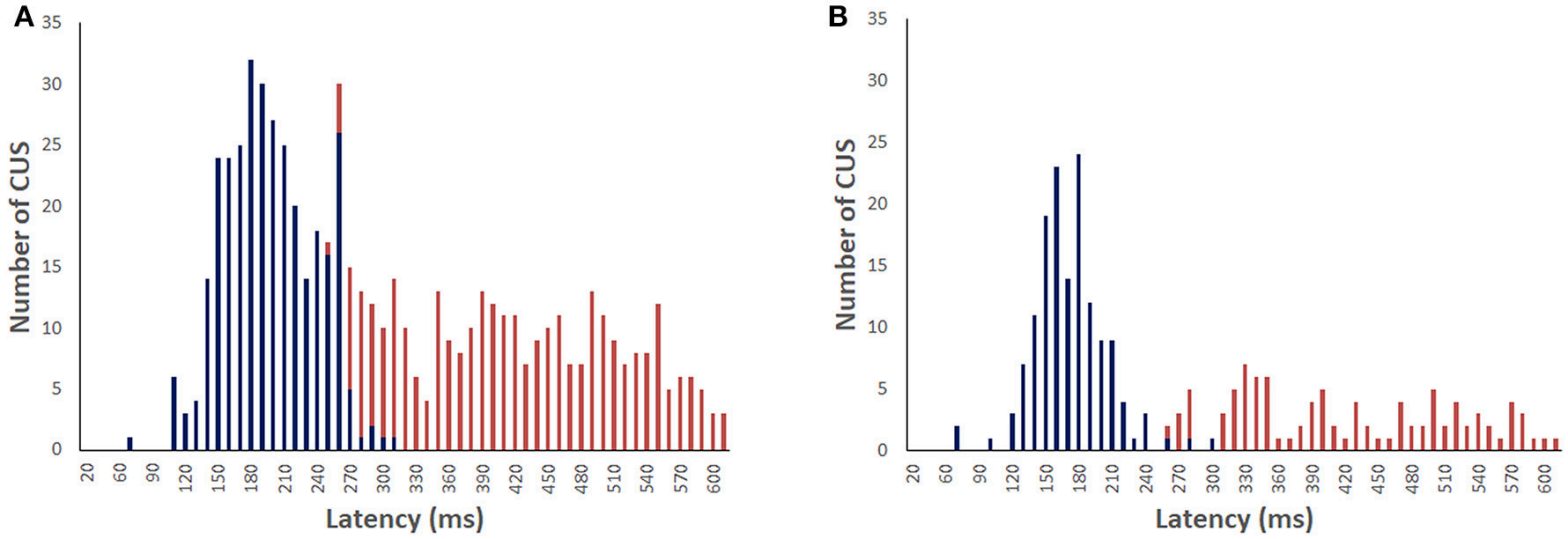
Distribution of the covert saccades (blue) and overt saccades (red) latencies in bilateral vestibular deficient group during vHIT recordings with light target **(A)** and in darkness with imaginary target **(B)**. The median of CUS peak velocity is 183 and 195 ms, respectively.

For the light-test, 96% of the head impulses were followed by CUS (*n* = 634). Figure 3A shows maximum velocity latency histogram of the first CUS (*n* = 317) for the recordings with visible target in light. The peak of the histogram is at 183 ms with onset latency about 20 ms earlier. The maximum velocity latency for all CUS showed a non-Gaussian distribution (Shapiro-Wilk W = 0.920 *p* < 0.0001) with a median at 183 ms. The median latencies of the first and subsequent CUS were similar:195, 171, 179, and 152 ms, respectively and the median latency, since onset of head impulse, of the second, third and fourth CUS range from 355 to 519 ms. For the vHIT in darkness (*n* = 241) the peak of maximum velocity latency of all CUS is 158 ms and the median is 195 ms (non-Gaussian distribution Shapiro-Wilk W = 0.857 *p* < 0.0001) (Figure 3B). There was a significant increase of the latency of all CUS in darkness compared to light (non-parametric test of Mann-Whitney *Z* = −4.975, *p* < 0.0001) but not for the first CUS (non-parametric test of Mann-Whitney *Z* = −0.319, *p* = 0.75), nor for the subsequent ones (non-parametric test of Mann-Whitney *Z* = −0,932, *p* = 0.35).

**Figure 4:**
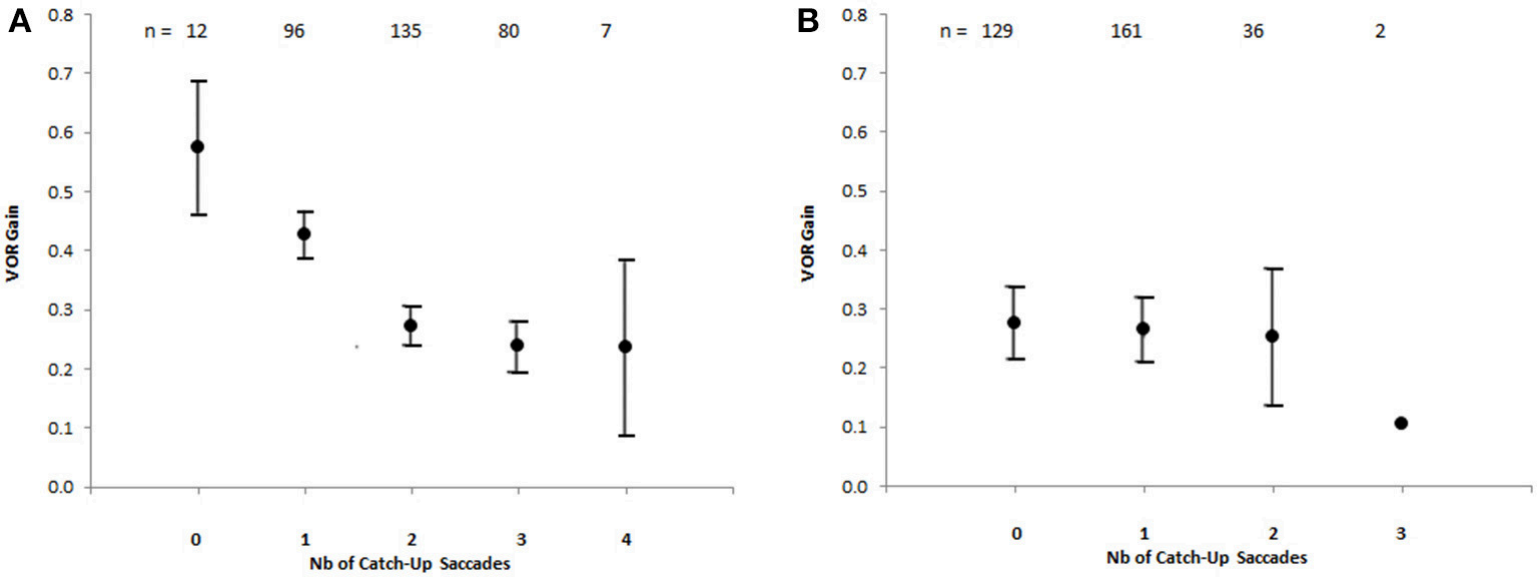
Number of catch-up saccades per record plotted against VOR gain in the bilateral vestibular deficient group, showing the increasing number of catch-up saccades with decreasing VOR gain value [Anova F_(4, 325)_ = 17,9 *p* < 0.00001] with visible target **(A)**. The vertical brackets represent 95% confidence interval. There is no similar relationship for recordings in darkness **(B)**. The values above indicate the number of records.

The number of CUS were plotted against the VOR gain values, showing that the number of CUS increased significantly as the gain value decreased in light-test [ANOVA F _(4, 325)_ = 17.9 *p* < 0.00001] but not in darkness (Figure 4).

**Figure 5:**
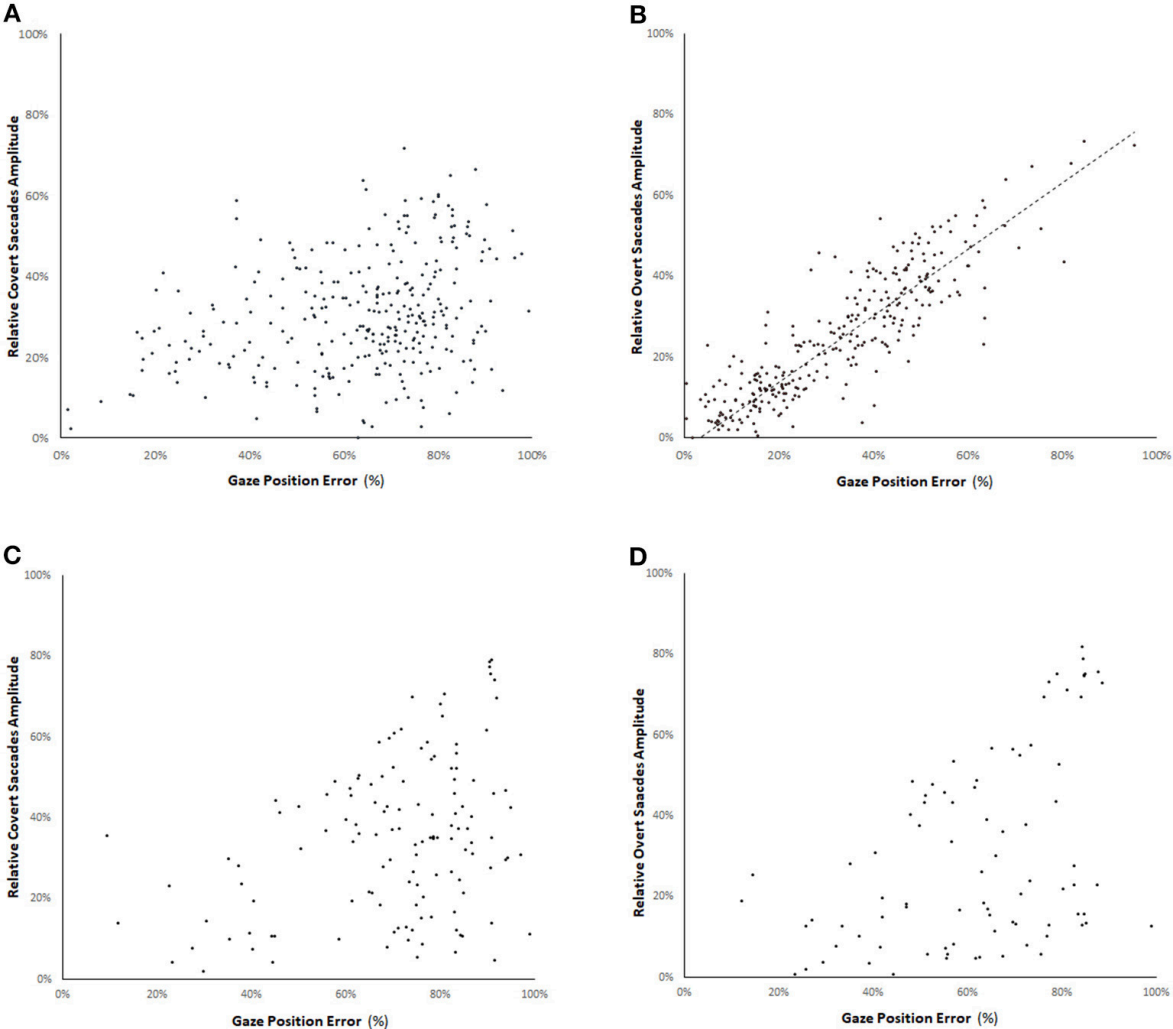
Relation between the relative amplitude of covert saccades **(A,C)** and overt saccades **(B,D)** (ratio between the amplitude of the CUS and the head rotation amplitude) and the gaze position error in the bilateral vestibular deficient group for vHIT with visible target **(A,B)** and in darkness with an imaginary target **(C,D)**. The gaze position error is the ratio between the remaining eye movement to reach the target (difference between the head rotation amplitude and the cumulative eye movement amplitude at the onset of the CUS) and the head rotation amplitude. There is a high correlation (straight line slope = 0.82, *r* = 0.79 *p* < 0.05) between the amplitude of the overt saccades and the gaze position error with visible target. The correlation coefficient of the amplitude of overt saccades in darkness is 0.55 **(D)**.

We assessed the relation between the relative amplitudes of covert and overt CUS (ratio between amplitude of the CUS and the head rotation amplitude) and the gaze position error (GPE). The gaze position error is the ratio between the remaining eye movement to reach the target (difference between the head rotation amplitude and the cumulative eye movement amplitude at the onset of the CUS) and the head rotation amplitude. Figure 5B shows a high correlation (*r* = 0.79 *p* < 0.05) between the amplitude of the overt saccades and the GPE in the light test. The correlation coefficient between the amplitude of the covert saccades and the GPE with visible target is 0.27 (*p* < 0.05).

In complete darkness, there is a drop of 62% of the number of CUS (241 in darkness vs. 634 with visible target). The mean reduction of saccade rate per record is 50% for the covert saccades and 65% for the overt saccades (Chi-squared = 6.58 *p* = 0.01) (Table 2). Furthermore, no clear relation could be noted between the relative amplitude of covert or overt saccades and the GPE for the amplitude of CUS performed in darkness-test (*R* = 0.33 and 0.55, respectively) (Figures 5C,D).

**Table 2:** The number of records, covert and overt saccades with light target and in darkness for each BVL patient.

**Patient**	**Visible target**			**Darkness**			**Differences of saccade rate per record**		
	**Records**	**Covert saccades**	**Overt saccades**	**Records**	**Covert saccades**	**Overt saccades**	**Covert saccades**	**Overt saccades**	**All CUS**
1	21	25	27	21	13	10	48%	63%	56%
2	24	24	38	21	1	3	95%	91%	93%
3	21	16	19	20	2	11	87%	39%	61%
4	19	12	12	22	2	9	86%	35%	60%
5	19	23	13	13	6	7	62%	21%	47%
6	19	17	20	19	2	12	88%	40%	62%
7	20	10	13	19	1	4	89%	68%	77%
8	19	25	15	22	7	8	76%	54%	68%
9	22	11	21	19	16	0	−68%	100%	42%
10	19	23	23	15	10	0	45%	100%	72%
11	17	22	11	17	9	8	59%	27%	48%
12	19	29	15	16	16	0	34%	100%	57%
13	14	10	11	15	8	1	25%	92%	60%
14	18	18	24	20	8	12	60%	55%	57%
15	20	15	11	24	5	2	72%	85%	78%
16	19	20	28	18	20	1	−6%	96%	54%
17	20	20	13	19	19	8	0%	35%	14%
Mean :							50%	65%	59%

*The difference of saccade rate per record in light and darkness is expressed in %. The mean difference of saccade rate per record for the whole group is 50% for covert saccades, 65% for overt saccades and 59 % for all CUS*.

In the subgroup of 6 BVL patients with VOR gain over 0.5, CUS were identified in 72 out of 80 recordings (90%) in light-test and in 35 out of 76 recordings (46%) in darkness-test (Chi-squared = 34.9 *p* < 0.001). The mean gains were 0.64 ± 0.12 and 0.4 ± 36, respectively (Student test for paired values, *p* < 0.001).

### Group of Patients With Normal vHIT Gain

For this group of 35 patients with normal vHIT we compared the VOR gain measured in light-test (*n* = 638) and in darkness-test (*n* = 615). The mean VOR gain for the entire group was 1.1 ± 0.14 (mean ± SD) in light-test and significantly reduced in darkness-test: 0.88 ± 0.24 (mean ± SD) (Student test for paired data *p* < 0.01). The occurrence of CUS was not significantly different in light-test and darkness-test: 9.4% vs. 8.1% (Chi-square = 0.63, *p* = 0.42) (data not shown).

## Discussion

These findings contribute understanding of parametric determinants of the compensatory CUS recorded during vHIT in BVL patients. Our results show significant changes of the CUS number, amplitude and latency after the suppression of visual cues in the group of BVL patients. This study suggests that visual input is the main trigger and determinant of the number, amplitude and latency of the CUS. We also confirm the hypothesis that a visible target increases the high-velocity VOR gain even in the control subjects with gains within the normal range (18).

### The Required Number of CUS Increases With Low VOR Gain

We found a mean onset latency of 163 ms for the first CUS. This is consistent with other results in the literature (5, 6, 8, 10). This time interval has been established as necessary and sufficient for a refixation saccade to be organized as substitution for a deficient VOR (19). Some authors have measured latencies as short as 70 ms with skewed distribution and the mean latency of 151 ms (6). One hypothesis is that these short latencies resulted from correctly anticipated head impulse. The latencies of CUS also increase with the decline of the head impulse acceleration (6).

CUS cannot be accurate if they occur during passive head movement because the end position cannot be predicted. Therefore, they are often followed by additional CUS. These can be hypothesized to be encoded after the end of head movement to fixate the gaze on target. When the head is immobile, the saccade should be accurate enough to put the eye position on target. Overt saccades are defined as occurring after the first moment at which the head velocity become zero (the null velocity point). The null velocity point is not equivalent to the end of head movement because it is often followed by a rebound movement in opposite direction. The mean latency of the head null velocity point in our series is 150 ms and the head is motionless at about 250 ms. In healthy subjects, saccades remain precise despite ongoing changes in head position in space (20). So, we can assume that patients with unilateral vestibular deficit remain qualified to generate accurate CUS on target position once the null velocity point is reached. During the possible following head rebound movement in the opposite direction, the target position is perceived as stable in space due to the efficient ipsilateral vestibular system. In case of bilateral vestibular deficit, the head has to be completely motionless before an accurate CUS could be generated. For visually guided saccades the delay between the target presentation and the start of the eye movement is about 180 ms (21). This may explain why in some of our bilateral vestibular deficient patients an accurate saccade cannot occur roughly before 430 ms. An additional time, likely due to the initial CUS, accounts for a median three or four CUS latency of 504 ms.

In this study, we also show that there is a significant relationship between the occurrence of multiple CUS and VOR gain. The number of CUS increases significantly with the drop in VOR gain, and thus with the gaze position error. Therefore, the amplitude of a single CUS, even programmed after the head movement does not systematically compensate a significant VOR gain deficit.

### Only Overt Saccades in Presence of Visual Target Are Efficient

Our study showed that the corrective amplitude of overt saccades is correlated with the GPE under visible target condition (Figure 5A). Similar relation was shown in a group of 8 patients with complete unilateral vestibular loss and one with BVL (6). Covert saccades are elicited by a velocity signal during the retinal slip. So, their amplitude cannot be determined by the residual distance to the target. Conversely, overt saccades are refixation saccades encoded based on a stationary GPE. During passive head movement of varying amplitude, the GPE could be based on residual vestibular information, on retinal inputs or on the weighing between cervical and oculomotor proprioceptive information. By suppressing retinal information concerning the target position we assessed the role of the visual information in processing the CUS. The similarities between CUS and head-fixed saccades mean sequence responses suggest that the CUS originate from the saccadic system (22). Saccade velocities were not included in our analysis because the maximum velocity of the CUS is determined by their amplitude (6).

### Less CUS in the Absence of Visible Target

Several reports reveal the high occurrence of CUS in unilateral and BVL (2, 6). In our BVL group, there is a significant drop (59%) in number of CUS in darkness-test suggesting that a visible target is a main factor for the CUS to supervene. Moreover, the lacking visual information induces the loss of relation between the residual CUS amplitude and the gaze position error (Figures 5B, D). This observation is in accordance with others (23) that showed an absence of CUS amplitude adaptation after reduction of VOR gain after a period of visual VOR suppression.

Literature data show that 1 year after neurectomy the ipsilateral VOR gain was 0.27+/-0.14 (1), suggesting that a gain over 0.5 is indicative of a residual vestibular function. A model proposed by Colagiorgio (24) hypothesize that covert saccades are driven by the prediction of head displacement using vestibular and extravestibular signals. For passive head impulses it is suggested that residual vestibular information may account for 80% of the estimated gaze position prediction. However, in our 6 patients with residual vestibular function the number of CUS also decreases significantly during the vHIT recordings performed without visible target despite the further reduction of the gain. Thus, the residual vestibular function in some patients or the inference of the gaze position error from cervical and oculomotor input are less efficient to generate adapted CUS. Nevertheless, the inter-individual variability of the CUS reduction in darkness could be explained by the use of proprioceptive triggers by some patients, especially those with lower residual VOR gain. In the presence of a residual vestibular function, the opening of the VOR loop in darkness impairs the triggering and adaptation of the CUS. Peng et al. (22) showed that corrective saccades can be generated in the absence of vision by flashing off the target when the head began to move. This is more suggestive of memorized target paradigm. Even in this condition, the authors observed that the CUS did not accurately minimize the GPE. We argue that the absence of a visual cues lowers the efficiency of the substituted saccadic system probably by opening the feed-back loop that controls the occurrence and accuracy of saccades.

### Visual Deprivation Lowers the VOR Gain

In both group of patients, our results showed significant decrease in VOR gain when no visual information about the target position was available. The modulation of normal VOR gain measured at high velocities by the vHIT was already addressed with variation of the gain by the target distance and the brightness of the peripheral visual field (18).

The incidence of CUS in normal subjects, measured by vHIT varies greatly in the literature, from 16.7 to 49% (7, 25). The CUS in normal subject probably compensate the hypometric characteristic of VOR, which increases with age (7). In our group with normal vHIT, we observed significant decrease of the VOR gain in darkness-test and the occurrence of CUS is 9.4 and 8.1% in light and dark conditions, respectively. The absence of significant increase in number of CUS in darkness-test despite the VOR gain reduction, could be explained by the lack of visual input.

The ocular pursuit system could be responsible for increased VOR gain with a visible target compared to dark condition. There is some evidence that the pursuit system is still necessary to enhance the VOR gain for large amplitude at low velocities (26). But, the smooth pursuit system has a latency of about 100 ms (27) and low velocities VOR gain significantly increase already during the initial 80 ms, when comparing VOR with visual fixation and in darkness (28). So, it seems unlikely that the pursuit system and the optokinetic system, which has a latency of 70 ms in humans (29), are able to increase the VOR gain during head thrusts that reach their peak velocities after about 90 ms. The target distance of 140 cm eliminates the vergence contribution during the target fixation. Attentiveness increases VOR gain (30), but we argue that the attention level do not significantly change from light condition to darkness with a precise task to imagine a visual target.

The efficiency of the VOR is powered by a visual feedback loop. Its main goal is to diminish the retinal image slip. This feedback loop modulates the activity of vestibular nuclei. This VOR gain modulation is an adaptive mechanism and the few minutes in light or darkness before the recording onset, followed by a set of at least ten recordings, allowed this mechanism to develop. Demer et al. (31) showed that VOR gain adaptation is already achieved 15 min after the wearing of magnifier spectacles, but an eventual adaptation for shorter time is not reported. Adaptation to the target distance can occur as early as 40 ms after the beginning of the head motion. (32). We argue that the VOR cannot be accurate without a constant modulation by the image stabilization feedback. The increase of VOR gain when the target is in light environment, opposite to dark environment (18) suggests that the VOR efficiency increases when an image has to be stabilized (23). We concur with Chim et al. (18) arguments in invoking the vestibular adaptation mechanisms to increase the high-frequency VOR response. The absence of oscillopsia passing from darkness to light suggests that this adaptation is a fast process. The retinal position error has been showed to increase the high-velocity VOR response (18). Conversely, the suppression of visual target opens the VOR arc, decreasing its efficiency. Similarly, to avoid the ocular pursuit interference, the low speed VOR is often evaluated in the absence of visual target. The large dispersion of normative values of VOR gain in these conditions is explained by the same mechanism (33). This raises the question about the reliability of VOR evaluation in the absence of target image on the retina.

## Conclusions and Perspectives

We found a drastic reduction in number of CUS under dark conditions, suggesting that the visual input is a main factor for a CUS to be generated, even in patients with residual vestibular function. The absence of visible target also reduces significantly the VOR gain and eliminates the relationship between the CUS amplitude and the remaining eye movement to compensate the passive head rotation.

The VOR appears to be a hypometric system (7) but the visual feedback information can modulate the VOR gain with a delay of 40 ms after the head movement (32). This short delay allows the adjustment of the VOR gain and CUS amplitude.

Further, studies are necessary for understanding the triggering of residual CUS in darkness and how CUS could bring supplementary improvement in rehabilitation techniques for the patients with vestibular deficiencies.

## Author Contributions

CVN, AB, UD, CH, and MT conceived and designed the experiments, wrote and revised the manuscript. CVN, AB, and UD performed the experiments. MT, CH, AB, and CVN recruited patients.

### Conflict of Interest Statement

The authors declare that the research was conducted in the absence of any commercial or financial relationships that could be construed as a potential conflict of interest.
